# Myasthenia gravis with thymoma-associated CRMP5/CV2 antibody-positive paraneoplastic neurological syndrome: a case report and literature review

**DOI:** 10.3389/fimmu.2026.1904729

**Published:** 2026-09-14

**Authors:** Rui Hu, Pankui Li, Yaping Jin, Qian Ma, Pei Li, Pan Yang, Zhenhai Wang

**Affiliations:** 1 First Clinical College of Ningxia Medical University, Yinchuan, China; 2 School of Nursing, Ningxia Medical University, Yinchuan, China; 3 Department of Neurology, General Hospital of Ningxia Medical University, Yinchuan, China; 4 Diagnosis and Treatment Engineering Technology Research Center of Nervous System Diseases of Ningxia Hui Autonomous Region, Yinchuan, China

**Keywords:** case report, CRMP5/CV2 antibody, immunotherapy, myasthenia gravis, paraneoplastic neurological syndrome, thymoma

## Abstract

**Objective:**

To delineate the clinical characteristics, diagnostic criteria, therapeutic strategies, and prognosis of myasthenia gravis (MG) with thymoma-associated CRMP5/CV2 antibody-positive paraneoplastic neurological syndrome (PNS), thereby enhancing clinical recognition of this rare overlapping autoimmune disorder.

**Methods:**

We retrospectively analyzed the clinical data of a 53-year-old male patient presenting with peripheral nerve injury as the initial manifestation, who was subsequently diagnosed with MG, invasive thymoma, and CRMP5/CV2 antibody-positive PNS. A comprehensive review of the relevant international literature was also conducted.

**Results:**

The patient had a 2-year disease course, initially presenting with limb numbness and unsteady gait, and gradually developing dysarthria, dysphagia, diplopia, and myasthenic weakness with characteristic diurnal fluctuation (worsening in the evening and improving in the morning). According to the Myasthenia Gravis Foundation of America (MGFA) clinical classification, the patient was categorized as Class IIIb (moderate generalized MG with predominant oropharyngeal involvement) at baseline. Serological tests confirmed positive for acetylcholine receptor (AChR) antibody in serum, as well as CRMP5/CV2 antibody in both serum and cerebrospinal fluid (CSF). Electrophysiological examinations revealed multiple peripheral nerve damage and a significant decremental response to low-frequency repetitive nerve stimulation (RNS). Chest CT demonstrated an invasive thymoma in the anterior mediastinum. The patient underwent thoracoscopic thymectomy, followed by sequential treatment with glucocorticoids, intravenous immunoglobulin and cyclophosphamide, as well as symptomatic supportive care and anti-infective management. After treatment, the neurological symptoms were significantly improved, and the condition remained stable during follow-up.

**Conclusion:**

MG with thymoma-associated CRMP5/CV2 antibody-positive PNS represents a rare clinical entity with atypical onset and is highly susceptible to misdiagnosis. Combined detection of paraneoplastic antibodies and chest imaging is critical for early diagnosis, and thymectomy combined with standardized immunotherapy can effectively improve the clinical symptoms and long-term prognosis of patients.

## 1 Introduction

Myasthenia gravis (MG) is an acquired antibody-mediated autoimmune disease primarily affecting the postsynaptic membrane of the neuromuscular junction. Thymoma is a major comorbidity of MG, and the two conditions share close immunopathological correlations (1). Paraneoplastic neurological syndromes (PNS) refer to remote neurological injuries triggered by humoral and immune responses underlying malignancies, rather than direct tumor infiltration, and are frequently accompanied by disease-specific neuronal autoantibodies (2). CRMP5/CV2 antibody is a relatively uncommon serological marker of PNS, which mainly damages the peripheral nerves, brainstem and central nerve conduction pathways (3).

The coexistence of thymoma-associated MG and CRMP5/CV2 antibody-positive PNS is extremely rare. The overlapping clinical manifestations of these two disorders, coupled with peripheral nerve dysfunction as the initial symptom, frequently lead to misdiagnosis as cervical spondylosis, idiopathic peripheral neuropathy, or other neurological disorders, ultimately resulting in delayed intervention. To date, relevant clinical case reports remain limited worldwide, and standardized diagnostic and therapeutic experience is lacking. Herein, we report a well-documented case with complete treatment procedures and long-term follow-up data. and further review the published literature to deepen the understanding of this rare disease and provide practical references for clinical management.

## 2 Case presentation

A 53-year-old male patient was admitted with aggravated limb numbness and unsteady gait with a chronic course of more than two years. At the end of 2023, he initially experienced persistent paresthesia in the left hand but did not seek timely medical attention. In January 2024, he developed swaying and unsteady walking, accompanied by numbness in both hands and feet. Thoracic spine MRI and lumbar MRI were performed, which showed no obvious abnormalities. Notably, the thoracic spine MRI did not include dedicated mediastinal sequences, and therefore the anterior mediastinum was not adequately assessed at that time. Electromyography (EMG) performed at that time also showed no obvious abnormalities. The patient received acupuncture treatment, with slight improvement in walking instability, whereas intractable limb numbness persisted. In July 2024, the numbness gradually aggravated and involved the bilateral forearms and lower legs. Walking dysfunction became progressively worse in November 2024, and generalized numbness occurred in January 2025. From March to April 2025, the patient gradually developed dysarthria, dysphagia, binocular diplopia, generalized myokymia and asymmetric palpebral fissures. His myasthenic weakness showed typical diurnal fluctuation, which deteriorated after activity and relieved after rest. Given the progressive symptoms, he was admitted to our department for further diagnosis and treatment.

The patient had a negative past medical history, and there was no family history of autoimmune diseases or hereditary neurological disorders. Neurological examination on admission revealed limited bilateral eye abduction and mild bilateral ptosis. The muscle strength of four limbs was basically normal. Hypoesthesia of superficial and deep sensation and decreased tendon reflexes were detected in the lower extremities, and bilateral pathological signs were negative. Serological testing confirmed positive for acetylcholine receptor (AChR) antibody in serum (titer 1:10) (**Figures 1A–C**), as well as CRMP5/CV2 antibody in both serum and cerebrospinal fluid (CSF) **Figures 2A–C**). Oligoclonal bands were absent in serum and CSF, with normal intrathecal immunoglobulin synthesis. Antibodies against CASPR2, gangliosides, nodal and paranodal proteins (NF155, NF186, CNTN1, CNTN2, CASPR1), and other paraneoplastic markers (HuD, Yo, Ri, Amphiphysin, Ma1, Ma2, SOX1, Tr, Zic4, GAD65) were all negative. Anti-MuSK antibody was also negative. Thyroid function and routine autoimmune indicators were within the normal range. Cerebrospinal fluid examination demonstrated normal intracranial pressure, cell count, protein, glucose and chloride levels, with no pathogenic pathogens detected and negative oligoclonal bands. According to the Myasthenia Gravis Foundation of America (MGFA) clinical classification, the patient was categorized as Class IIIb (moderate generalized MG with predominant oropharyngeal and respiratory muscle involvement, accompanied by ocular muscle weakness) (4). The baseline modified Rankin Scale (mRS) score was 3, indicating moderate disability with requirement for some help with daily activities, but with no need for continuous care (5). Electrophysiological tests confirmed multiple peripheral nerve injuries, predominantly sensory axonal damage, and abnormal conduction of somatosensory, visual and brainstem auditory evoked potentials. Low-frequency repetitive nerve stimulation presented a distinct decremental response. Chest CT scanned on June 23, 2025 revealed an irregular solid mass measuring 5 cm × 4 cm × 3 cm in the anterior superior mediastinum, which was highly considered as invasive thymoma closely adhering to the pericardium and left lung tissue (**Figures 3A, B**). Thoracic spine MRI only showed mild degenerative changes without spinal cord compression. The patient was transferred to the thoracic surgery department and underwent thoracoscopic mediastinal lesion resection combined with extended thymectomy under general anesthesia. Postoperative pathological and immunohistochemical results were as follows: TdT (+), CD1a (+), CD5 (+), CKpan (+), P40 (+), Ki-67 index of approximately 70%, negative CD117 and NUT, intact expression of BRG1 and INI-1, and focal CD20 positivity. Combined with histological morphology, the lesion was pathologically diagnosed as type B2 thymoma (**Figures 4A–C**). In view of tumor invasion into the pericardium and left lung, as well as dense adhesion to the left innominate vein and phrenic nerve, the tumor was staged as Masaoka stage III. Postoperative chest CT re-examination on July 2, 2025 showed postoperative changes in the anterior superior mediastinum with blurred fat space in the surgical area (**Figures 3C, D**). Follow-up CT on August 4, 2025 showed significant imaging improvement compared with previous scans (**Figures 3E, F**). Chest CT on March 13, 2026 indicated a favorable postoperative outcome without tumor recurrence (**Figures 3G, H**).

**Figure 1:**
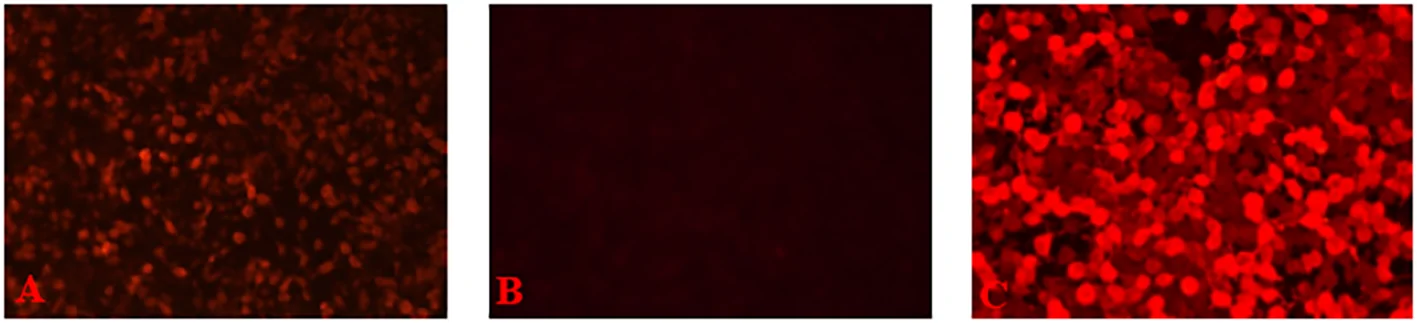
Cell-based assay (CBA) for anti-acetylcholine receptor (AChR) antibody detection. Patient serum IgG binding (red fluorescence) **(A)**. Negative control with no specific IgG binding **(B)** Positive control showing strong IgG reactivity on AChR-transfected cells **(C)**.

**Figure 2:**
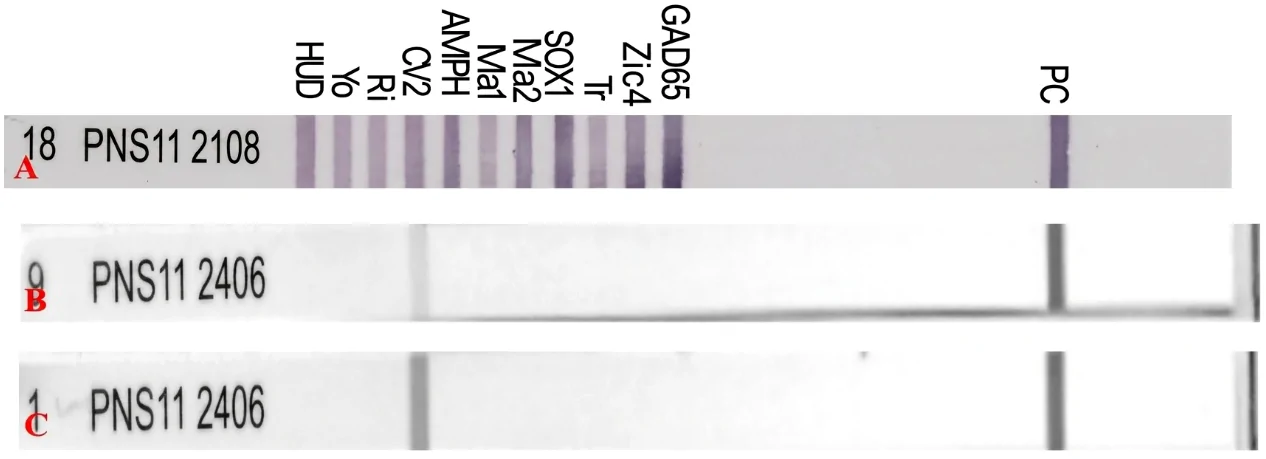
Immunoblot analysis of paraneoplastic antibodies in serum and cerebrospinal fluid (CSF). Position reference strip showing the antigen array layout, including HuD, Yo, Ri, CV2, Amphiphysin, Ma1, Ma2, SOX1, Tr, Zic4, GAD65, and positive control (PC) **(A)**. CSF immunoblot showing weakly positive reactivity for anti-CV2 (CRMP5) antibody IgG (strip labeled “9”, PNS11 2406) **(B)**. Serum immunoblot demonstrating positive reactivity for anti-CV2 (CRMP5) antibody IgG (strip labeled “1”, PNS11 2406) **(C)**. Note the absence of reactivity for other paraneoplastic antibodies in both serum and CSF.

**Figure 3:**
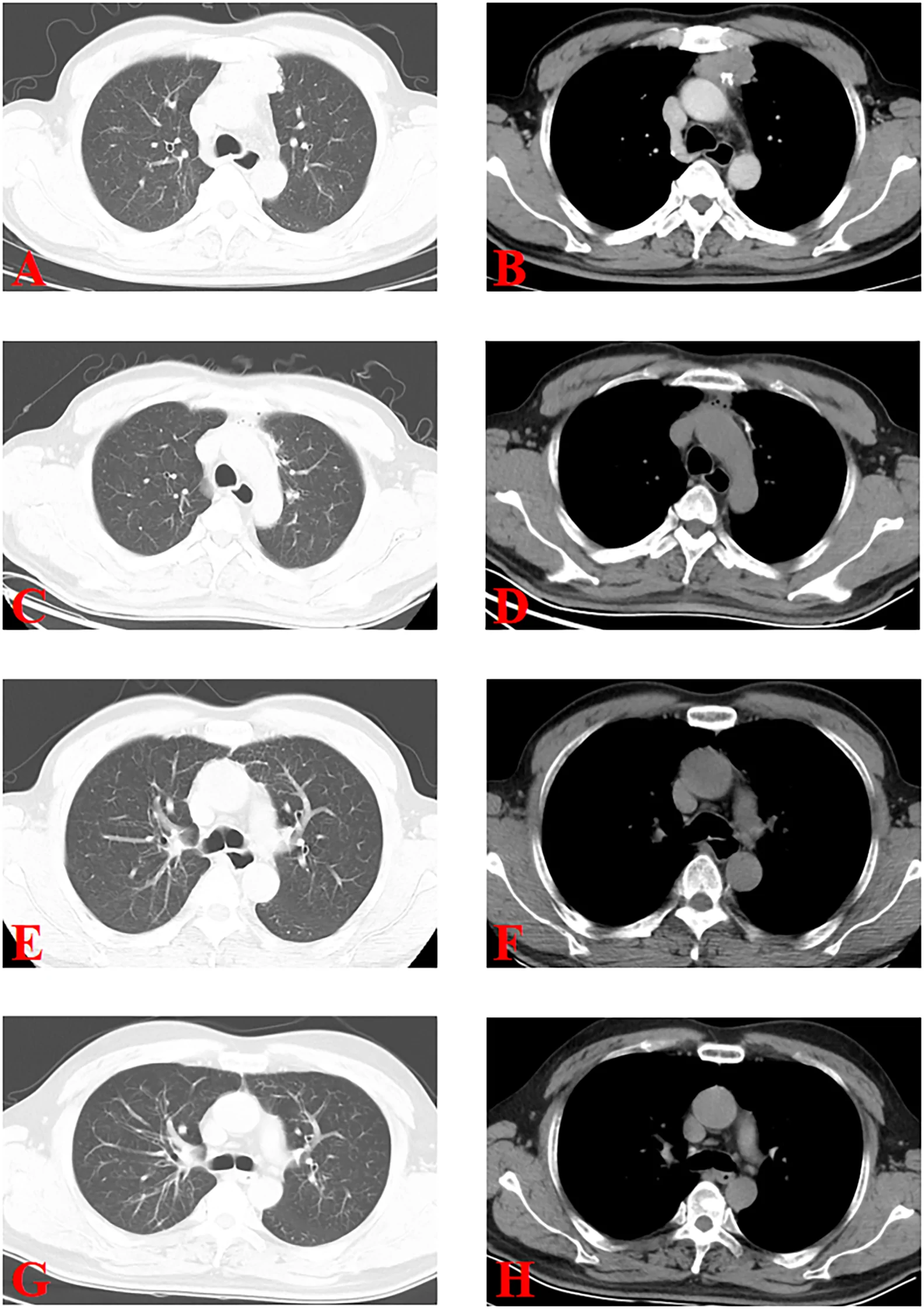
Chest CT scans obtained on June 23, 2025 revealed an irregular solid mass in the anterior superior mediastinum, highly suggestive of invasive thymoma **(A, B)**. Postoperative chest CT re-examination on July 2, 2025 showed postoperative changes of the anterior superior mediastinum with blurred fat spaces in the surgical region **(C, D)**. Follow-up CT on August 4, 2025 demonstrated marked radiographic improvement compared with prior images **(E, F)**. Chest CT acquired on March 13, 2026 confirmed a favorable postoperative outcome with no signs of tumor recurrence **(G, H)**.

**Figure 4:**
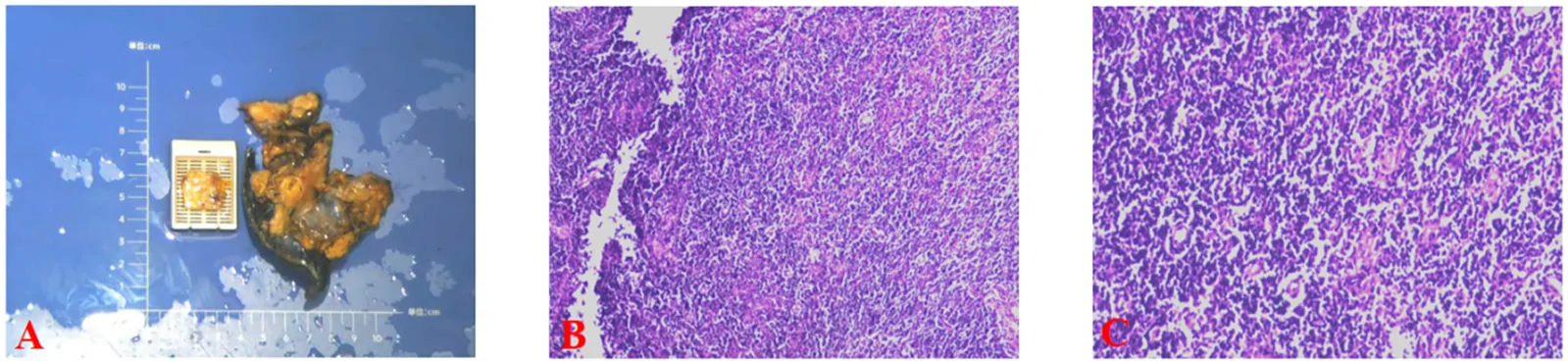
Gross and histopathological findings of the resected thymoma. Gross surgical specimen of the resected invasive thymoma, with a centimeter-scale ruler for size reference **(A)**. Low-power view of hematoxylin-eosin (H&E)-stained histologic section, showing the typical architectural features of type B2 thymoma (×100) **(B)**. High-power view of H&E-stained section, demonstrating the classic morphology of type B2 thymoma: scattered neoplastic epithelial cells admixed with abundant immature T lymphocytes (×200) **(C)**.

Integrating clinical symptoms, physical signs and auxiliary examinations, the final diagnoses were confirmed: (1) Moderate generalized myasthenia gravis with positive AChR antibody; (2) Invasive thymoma; (3) CRMP5/CV2 antibody-positive paraneoplastic neurological syndrome.

During the postoperative period, the patient developed worsening dysarthria and dysphagia in August 2025 compared with the preoperative condition and was admitted with the diagnosis of paraneoplastic neurological syndrome (PNS). After admission, intravenous human immunoglobulin was infused at 0.4 g/kg/day for 5 consecutive days for immunomodulation targeting CV2-antibody-positive PNS. For moderate-generalized acetylcholine receptor (AChR)-positive myasthenia gravis, intravenous methylprednisolone sodium succinate 40 mg once daily was administered for 5 days, together with oral pyridostigmine bromide 60 mg three times daily. The patient achieved subjective symptomatic improvement and was discharged. After discharge, he was maintained on regular oral prednisone acetate 40 mg once daily and pyridostigmine bromide 60 mg three times daily.

Twenty-five days after discharge, the patient reported aggravated limb numbness accompanied by ptosis and gait instability, and was readmitted. High-dose intravenous glucocorticoid pulse therapy was performed: methylprednisolone 1000 mg daily for 3 days, followed by 500 mg daily for 3 days, 240 mg daily for 3 days, and 120 mg daily for 3 days. Therapy was then switched to oral prednisone acetate 60 mg (12 tablets) administered at 7-8 a.m. once daily. The dose was tapered by 10 mg (2 tablets) per week until reaching 30 mg daily, which was sustained for 2 weeks; thereafter, the dose was reduced by 5 mg (1 tablet) every 2 weeks until complete discontinuation.

In October 2025, the patient visited the Department of Radiation Oncology for radiotherapy. The clinical target volume (CTV) encompassed the mediastinal tumor bed, and the planning target volume (PTV) was generated by uniformly expanding the CTV by 0.3 cm. The WAT irradiation technique was adopted, and 50 Gy in 25 fractions of 2.0 Gy was prescribed to 95 % of the PTV. After 4 fractions of radiotherapy (not chemotherapy), the patient presented with choking cough and increased sputum production. Repeat chest CT revealed aggravated bilateral pneumonia, which was considered to be secondary to glossopharyngeal nerve palsy induced by myasthenia gravis. The patient received anti-infective treatment in a local hospital. During that hospitalization, pulmonary infection complicated with Mycobacterium tuberculosis infection occurred, which was effectively controlled by targeted anti-infective therapy.

The patient was re-admitted to our department on October 31, 2025. For myasthenia gravis, intravenous immunoglobulin pulse therapy (0.4 g/kg/day for 5 consecutive days) was given, combined with intravenous cyclophosphamide 400 mg. Subsequently, the patient received long-term treatment with oral pyridostigmine bromide 60 mg three times daily plus intravenous cyclophosphamide 400 mg every two weeks.

At the last follow-up in April 2026, the patient could climb stairs independently but exhibited mild weakness of bilateral lower extremities; he required support when squatting and standing up. Vital signs were as follows: body temperature 36 °C, respiratory rate 20 breaths/min, blood pressure 122/84 mmHg. Neurological examination showed clear consciousness and fluent speech. Bilateral ocular abduction was limited with 1-mm scleral show, and nystagmus was absent. Bilateral pupils were isocoric with a diameter of approximately 3 mm; direct and indirect pupillary light reflexes were brisk. Bilateral masticatory strength was normal with full mouth opening, and bilateral corneal reflexes were intact. Bilateral eye closure was mildly weak. Muscle strength of all four limbs was grade 5, and limb muscle tone was normal. Patellar clonus, ankle clonus, bilateral Hoffmann’s sign, Rossolino’s sign, Babinski’s sign and Chaddock’s sign were all negative. No other remarkable abnormal neurological findings were identified. The Activities of Daily Living (ADL) score was 3 points (diplopia 1, respiratory function 1, lower-extremity function 1). The patient achieved MGFA post-intervention status of minimal manifestations (MM), with an mRS score of 1, indicating only minor residual symptoms without significant functional disability. Chest CT showed no evidence of tumor recurrence, and the patient was considered to be in complete oncological remission. Follow-up serological testing for AChR antibody and CRMP5/CV2 antibody was not re-evaluated due to limited resources, which represents a limitation of this study. Up to April 2026, the patient remained stable during long-term follow-up, without obvious disease recurrence or severe treatment-related adverse reactions (**Table 1**).

**Table 1:** Timeline of clinical presentation, diagnostic findings, therapeutic interventions, and outcomes.

Phase	Time	Clinical manifestations & diagnostic findings	Therapeutic interventions	Outcomes & key metrics
1. Symptomatic Onset & Progression	Late 2023 – May 2025	Symptoms: Progressive limb paresthesia, gait ataxia, followed by dysarthria, dysphagia, diplopia, and diurnal fluctuating weakness. Initial EMG (Jan 2024): Normal.	Symptomatic management (e.g., acupuncture) with limited efficacy.	Progressive neurological decline.
2. Diagnostic Workup	Jun– Jul 2025	Neurological Exam (May 2025): MGFA Class IIIb; mRS 3. Serology: AChR Ab positive (titer 1:10); CRMP5/CV2 Ab positive (serum & CSF). Electrophysiology: Sensory axonal peripheral neuropathy; Decremental response on low frequency RNS. Imaging: Chest CT revealed anterior mediastinal mass (5×4×3 cm). Pathology (post op): Type B2 thymoma, Masaoka stage III (invading pericardium & lung).	Surgical: Extended thymectomy (thoracoscopic).	Etiology confirmed. High risk tumor with complete macroscopic resection achieved.
3. Multimodal Immunotherapy & Complications	Aug –Nov 2025	PNS/MG Exacerbation: Post operative worsening of bulbar symptoms and pneumonia. Infection: Complicated by pulmonary Mycobacterium tuberculosis.	Immunotherapy: Corticosteroids (high dose pulse + oral taper), IVIG (repeated cycles), and Cyclophosphamide (IV). Supportive: Anti infective therapy for tuberculosis. Adjuvant: Radiotherapy (50 Gy/25 fractions, interrupted).	Infection controlled. Immunotherapy transitioned to maintenance phase.
4. Long Term Follow Up & Remission	Nov 2025 – Apr 2026	Neurological: Residual mild bilateral lower limb weakness and paresthesia. Imaging (Mar 2026): No tumor recurrence. Antibodies: Not re tested (resource limitation).	Maintenance: Oral pyridostigmine + IV Cyclophosphamide (bi weekly).	MGFA: Minimal Manifestations (MM). mRS: Improved from 3 to 1. ADL Score: 3 (diplopia 1, respiration 1, lower limbs 1). Oncological: Complete remission.

## 3 Discussion and literature review

Myasthenia gravis (MG) is an acquired autoimmune disorder characterized by muscle weakness and fatigability, which is predominantly mediated by autoantibodies targeting components of the neuromuscular junction, with the acetylcholine receptor (AChR) serving as the most common antigenic target. The pathogenesis of MG is closely associated with thymic abnormalities, among which thymic hyperplasia and thymoma are the major associated lesions (6). Thymic alteration acts as a crucial pathogenic factor in a subset of MG patients, and accumulating evidence indicates that the tumor microenvironment of thymoma facilitates the generation and proliferation of autoreactive lymphocytes. Beyond its intimate association with MG, thymoma has been increasingly recognized to trigger a broad spectrum of systemic autoimmune diseases. The dysregulated immune microenvironment within thymoma disrupts central and peripheral immune tolerance, driving the production of autoantibodies against diverse neuronal and non-neuronal antigens (7). This immunological disturbance accounts for the frequent coexistence of MG and paraneoplastic neurological syndromes (PNS). PNS represents a heterogeneous group of immune-mediated disorders caused by cross-reactive immune responses against tumor antigens and homologous neural antigens, rather than direct tumor invasion or metastasis.

Among well-characterized onconeural antibodies, CV2 antibody targeting collapsin response mediator protein 5 (CRMP5) possesses critical diagnostic significance. CRMP5 is a cytoplasm-enriched protein highly expressed in neurons, which participates in axon guidance and neuronal differentiation (8). Seropositivity for CRMP5/CV5 antibody is a classic serological biomarker of PNS and strongly indicates an underlying malignancy, with thymoma and small cell lung cancer being the most frequently correlated tumors. CRMP5/CV2 antibody-positive PNS exhibits prominent clinical heterogeneity and can involve any segment of the neural axis, including the cerebrum, cerebellum, brainstem, spinal cord, dorsal root ganglia, peripheral nerves, and even the neuromuscular junction. Consequently, its clinical manifestations present complicated and overlapping features with MG, leading to frequent differential diagnostic difficulties (9).

Of particular note, the initial EMG examination performed in January 2024 revealed no obvious abnormalities, despite the patient’s progressive sensory symptoms. This finding is not uncommon in the early stages of paraneoplastic peripheral neuropathy. Several factors may account for the initially normal EMG results. First, in the early phase of PNS-associated peripheral nerve injury, pathological changes may be predominantly confined to small-fiber or sensory nerve terminals, which are not readily detectable by conventional EMG and nerve conduction studies (NCS) that primarily assess large myelinated fibers (10). Second, the pathophysiological process in CRMP5/CV2 antibody-associated neuropathy may initially involve immune-mediated axonal dysfunction rather than structural axonal loss, which may precede electrophysiological abnormalities by weeks to months (11, 12). Third, the predominantly sensory and axonal nature of the neuropathy in this case, as confirmed by subsequent electrophysiological studies, may have been below the detection threshold of standard EMG protocols during the initial evaluation. This observation underscores the importance of serial electrophysiological monitoring in patients with suspected PNS and progressive neurological symptoms, and highlights the risk of false reassurance from a single normal EMG study in the context of a rapidly evolving paraneoplastic process.

The coexistence of myasthenia gravis (MG), thymoma, and CV2/CRMP5 antibody-positive paraneoplastic neurological syndrome (PNS) constitutes a rare but clinically significant overlap syndrome. A retrospective study of patients with serum anti-CV2 antibody positivity identified thymoma as one of the most common associated malignancies, indicating a specific immunological correlation between the two entities (13). The core mechanism underlying this association stems from the thymus’s central role in the immune system: thymoma disrupts the normal selection and maturation of T cells, leading to the generation of autoreactive T cells and subsequent induction of multiple autoantibodies, including those targeting neuronal antigens such as CV2/CRMP5 (14). This overlap syndrome predominantly affects middle-aged adults with no obvious gender preference; a cohort study reported a mean patient age of 54 years and a male predominance (13). Pathologically, thymomas associated with this triad are mostly type B, with type B2 and B3 subtypes being the most common (14). These subtypes are characterized by higher lymphocyte infiltration and more prominent epithelial cell proliferation, correlating with their stronger immunogenicity and potential to elicit various paraneoplastic autoantibodies—not limited to CV2/CRMP5, but also including other types such as anti-AMPAR antibodies (15, 16). An important clinical observation indicates that CV2/CRMP5 antibody positivity can emerge before the definitive diagnosis of thymoma, and even prior to the onset of typical MG symptoms, serving as a crucial serological warning signal for occult tumors (17). This is exemplified by a juvenile case of paraneoplastic limbic encephalitis, where thymoma was diagnosed several years after the detection of anti-CV2/CRMP5 antibodies, and the antibody became undetectable following thymectomy. Thus, in patients with neurological symptoms, even if no known tumor is identified, the detection of CV2/CRMP5 antibodies should prompt immediate comprehensive screening for thymoma, as this antibody acts as a vital early diagnostic marker for such occult tumors (9).

The diagnosis of PNS relies on detecting high-titer specific antibodies in serum and/or cerebrospinal fluid (CSF), the gold standard for diagnostic confirmation (18). The presence of CV2 antibodies in the CSF, particularly when accompanied by pleocytosis (predominantly lymphocytic) or elevated protein levels, strongly supports intrathecal antibody synthesis and indicates active immune-mediated inflammation within the central nervous system (CNS) (19, 20). For instance, in cases of paraneoplastic myelopathy, CSF analysis typically reveals lymphocytic pleocytosis with elevated protein, which, combined with positive antibody results, confirms the diagnosis (20). Additionally, concurrent detection of anti-CV2 and anti-recoverin antibodies has been reported in patients with multiple paraneoplastic manifestations (21). Neuroimaging also plays a pivotal role in characterizing the neurological involvement of CV2 antibody-associated PNS. In CV2-related limbic encephalitis, brain magnetic resonance imaging (MRI) demonstrates hyperintensities on T2-weighted/fluid-attenuated inversion recovery (FLAIR) sequences in the medial temporal lobes, involving the amygdalae and hippocampi (19). Cerebellar involvement, presenting as ataxia, may be accompanied by cerebellar atrophy on imaging (3). When structural MRI results are inconclusive or unremarkable, [18F]fluorodeoxyglucose positron emission tomography/computed tomography ([18F]FDG-PET/CT) serves as a valuable supplementary modality. FDG-PET can detect increased metabolism in the limbic system earlier than MRI, and simultaneously enables systemic tumor screening—making it an essential tool for identifying occult malignancies such as thymoma or evaluating the metabolic activity of known thymomas (22). For the concurrent diagnosis of MG, repetitive nerve stimulation (RNS) and single-fiber electromyography (SFEMG) are key examinations to confirm neuromuscular transmission dysfunction. To evaluate potential peripheral nerve involvement (e.g., paraneoplastic sensory neuronopathy), nerve conduction studies (NCS) and conventional electromyography (EMG) can be employed (3). Integrating the results of neuroimaging, electrophysiological examinations, and serological data is critical for localizing neurological lesions, assessing the severity of damage, and guiding differential diagnosis.

Clarifying the underlying etiology and pathological characteristics of thymoma is essential for the standardized management of patients with myasthenia gravis (MG) complicated by CV2 antibody-positive paraneoplastic neurological syndrome (PNS). Contrast-enhanced chest computed tomography (CT) is the preferred first-line imaging modality for the detection, localization and preliminary radiological evaluation of thymic masses (22). For patients with high clinical suspicion of a paraneoplastic origin but negative or inconclusive CT findings, further examinations, including chest magnetic resonance imaging (MRI) and whole-body positron emission tomography-computed tomography (PET-CT), should be performed to identify occult tumors (22). Once thymic lesions are detected, histopathological confirmation via percutaneous biopsy or radical surgical resection is critical for definitive diagnosis. Pathological subtyping of thymoma in accordance with the World Health Organization (WHO) classification is of great clinical significance, since distinct histological subtypes exhibit varied risks of inducing autoimmune responses, autoantibody production and secondary neurological complications. Comprehensive pathological assessment of surgical specimens not only confirms the primary disease but also provides reliable prognostic evidence. A complex case of recurrent thymoma-related paraneoplastic encephalitis has validated this viewpoint, in which residual thymic tissue contributed to the production of novel autoantibodies and recurrent neurological deficits, necessitating secondary surgical resection (23). In two reported cases of autoimmune sarcoidosis with anti-contactin 1 (CNTN1) antibodies and concomitant thymoma, thymectomy failed to improve neurological symptoms, suggesting that therapeutic responses vary considerably across different autoimmune phenotypes. Regular postoperative chest CT surveillance is therefore recommended to monitor local recurrence or distant metastasis, as the long-term recurrence rate of thymoma ranges from 10% to 30% (24). During long-term follow-up, close monitoring of adverse reactions associated with chronic immunosuppressive therapy is also indispensable for ensuring treatment safety.

From the patient’s perspective, the long-term prognosis of this rare overlap syndrome is determined by both oncological control and neurological recovery. In the present case, the patient reported substantial improvement in health-related quality of life following comprehensive treatment. Prior to intervention, the patient was unable to perform routine daily activities independently due to combined peripheral neuropathy and myasthenic weakness. At the 10-month follow-up, neurological examination demonstrated near-complete functional recovery: muscle strength of all four limbs was grade 5, speech and swallowing were fluent, and the patient had returned to independent living. Only minor residual deficits persisted, including limited bilateral eye abduction with 1-mm scleral show, mild weakness of eye closure, and mild paresthesia of both lower extremities. Notably, bilateral lower extremity pitting edema was observed, which was considered likely related to chronic glucocorticoid use and reduced physical activity, rather than direct neurological involvement. The Activities of Daily Living (ADL) score was 3 (diplopia 1, respiratory function 1, lower-extremity function 1), and the modified Rankin Scale (mRS) improved from 3 (moderate disability) at baseline to 1 (minor symptoms without disability) at last follow-up, reflecting a clinically meaningful functional recovery. The maintenance immunosuppressive regimen was well tolerated,. Notably, patients with thymoma-associated CV2/CRMP5 antibody-positive PNS generally demonstrate a more favorable prognosis compared to those with small cell lung cancer (SCLC), primarily due to younger age at onset, lower malignancy potential of thymoma, and the reversibility of MG-specific symptoms following thymectomy and immunotherapy (25). However, the persistence of CRMP5/CV2 antibodies and the potential for late-onset neurological deterioration necessitate prolonged immunosuppressive therapy and vigilant long-term monitoring.

In-depth mechanistic research is urgently required to elucidate the precise cellular and molecular mechanisms within the thymoma microenvironment that initiate and maintain CRMP5-specific autoimmune responses. The application of clinical thymoma tissues and the construction of disease-simulating animal models will help clarify the regulatory roles of tumor-infiltrating lymphocytes, antigen-presenting cells and cytokine networks in breaking immune tolerance (26, 27). Apart from serial autoantibody titers, more sensitive and dynamic biomarkers for evaluating disease activity and treatment response are urgently needed. Potential candidates include phenotypic profiling of T-cell subsets (effector T cells, memory T cells and regulatory T cells), quantitative detection of cytokine and chemokine signatures in serum and cerebrospinal fluid, and measurement of neuroaxonal injury markers such as neurofilament light chain (NfL), which has been proven to possess promising prognostic value in multiple autoimmune neurological diseases (28–30). High-quality prospective clinical trials are needed to optimize treatment regimens and compare the efficacy and safety of diverse immunosuppressive protocols and novel biological agents, including complement inhibitors and B-cell depletion therapy, for thymoma-associated CV2-positive PNS (31, 32). Further research should focus on developing precise targeted immunotherapies to regulate specific immune pathways without causing extensive systemic immunosuppression (27). Preventive strategies also deserve systematic exploration. It is necessary to evaluate the cost-effectiveness and clinical utility of routine CV2 antibody screening in all patients diagnosed with MG and thymoma, even in the absence of obvious neurological manifestations. Moreover, whether early immunomodulatory intervention for asymptomatic antibody-positive individuals can prevent the onset of severe paraneoplastic neurological disorders and alter the natural disease course remains to be verified by high-level clinical evidence (27, 33).

Currently, most relevant studies on MG combined with thymoma-related CV2 antibody-positive PNS are limited to sporadic case reports, while large-sample controlled studies are still lacking. Existing diagnostic and therapeutic strategies are mainly summarized from empirical clinical practice. For patients presenting with unexplained peripheral nerve lesions combined with fluctuating myasthenic weakness, clinicians should actively complete paraneoplastic antibody profiling, MG-specific serological tests and chest imaging examinations at an early stage, so as to avoid missed diagnosis of underlying thymoma and occult paraneoplastic immune damage.

## 4 Conclusions

Myasthenia gravis (MG) with thymoma-associated CV2 antibody-positive paraneoplastic neurological syndrome (PNS) is a rare autoimmune overlap syndrome characterized by atypical clinical manifestations and is highly susceptible to misdiagnosis or delayed diagnosis. Combined detection of autoantibodies and chest imaging examinations is the key to early definite diagnosis. The comprehensive therapeutic regimen of thymectomy combined with glucocorticoids, intravenous immunoglobulin and immunosuppressants has proven efficacy, which can significantly improve patients’ clinical symptoms and long-term prognosis. Due to the rarity of this disease and limited clinical reports, this case report and the accompanying literature review can provide references for the diagnostic and therapeutic strategies of similar patients, and also offer clinical basis for further mechanistic research on this disease.

## Data Availability

The original contributions presented in the study are included in the article/supplementary material, further inquiries can be directed to the corresponding author.
